# Rocky Mountain spotted fever is a neglected tropical disease in Latin America

**DOI:** 10.1371/journal.pntd.0012276

**Published:** 2024-07-11

**Authors:** Gerardo Álvarez-Hernández, Christopher D. Paddock, David H. Walker, Jesus G. Valenzuela, J. R. Tadeo Calleja-López, Cristian Noé Rivera-Rosas, Rogerio Rafael Sotelo-Mundo

**Affiliations:** 1 Department of Medicine and Health Sciences, University of Sonora, Hermosillo, Sonora, Mexico; 2 National Center for Emerging and Zoonotic Infectious Diseases, Centers for Disease Control and Prevention, Atlanta, Georgia, United States of America; 3 Department of Pathology, University of Texas Medical Branch, Galveston, Texas, United States of America; 4 Vector Molecular Biology Section, Laboratory of Malaria and Vector Research, National Institute of Allergy and Infectious Diseases, National Institutes of Health, Rockville, Maryland, United States of America; 5 Centro de Investigación en Alimentación y Desarrollo A.C., Hermosillo, Sonora, México; Baylor College of Medicine, UNITED STATES

## Abstract

Rocky Mountain spotted fever (RMSF), a severe and extraordinarily lethal infectious disease, has emerged as a widespread public health crisis among predominantly vulnerable populations in several countries of Latin America, particularly evident in northern Mexico. Historically, RMSF has gained less attention than many other tropical infectious diseases, resulting in insufficient allocations of resources and development of capabilities for its prevention and control in endemic regions. We argue that RMSF fulfills accepted criteria for a neglected tropical disease (NTD). The relative neglect of RMSF in most Latin American countries contributes to disparities in morbidity and mortality witnessed in this region. By recognizing RMSF as an NTD, an increased public policy interest, equitable and more appropriate allocation of resources, scientific interest, and social participation can ameliorate the impact of this potentially treatable disease, particularly in vulnerable populations.

## Author summary

Rocky Mountain spotted fever (RMSF), a severe and extraordinarily lethal infectious disease, affects predominantly vulnerable populations in several countries of Latin America. The pathogen, *Rickettsia rickettsii*, is transmitted to humans through the bite of infected hard ticks from the family Ixodidae. When RMSF is not diagnosed or treated promptly, it often results in life-threatening disease and can be rapidly fatal in as many as 30% to 60% of patients. Among survivors, RMSF can result in long-term sequelae, including permanent cognitive deficits and amputations resulting from gangrene of extremities and appendages.

Hyperendemic levels of RMSF, witnessed currently in multiple states across northern Mexico, arises from a complex interaction of zoonotic, environmental, and social determinants that pose enormous challenges to health systems and public policies. These same determinants also exist in most Latin American countries, which place the region at great risk for devastating levels of disease. Historically, RMSF has gained far less attention than many other tropical infectious diseases of equivalent or lesser magnitude, resulting in insufficient allocation of resources and limited efforts for its prevention and control. The neglect of RMSF in most Latin American countries contributes to broadening disparities in morbidity and mortality across this region that involve predominantly persons living in poverty, children, and to an increasing degree, pregnant women. Formal recognition of RMSF as a neglected tropical disease is an important step toward reducing the public health burden created by this disease, fostering political and scientific interest and increasing financial resources for the implementation of medical and sanitary interventions.

## Introduction

Rocky Mountain spotted fever (RMSF), a tick-borne infectious disease (TBDs) caused by the gram-negative bacterium *Rickettsia rickettsii*, is transmitted by hard ticks of the genera *Dermacentor*, *Haemaphysalis*, *Rhipicephalus*, and *Amblyomma* in the family Ixodidae. Until the beginning of the 21st century, RMSF was considered a rare and sporadic infection in most countries of the Western Hemisphere. For reasons that remain unclear, RMSF reemerged during the last 25 years in multiple communities of the southwestern United States, and even more dramatically, across several towns and cities in several states of northern Mexico, where it now exists as hyperendemic disease in peri-domestic settings [1–3]. The public health implications of a distinct zoonotic cycle involving free-roaming and stray dogs that support enormous populations of the vector tick species, *Rhipicephalus sanguineus sensu lato*, are profound. RMSF is a life-threatening disease that is no longer rare or sporadic in these regions.

Historically, high frequencies of fatal RMSF have been recorded throughout the Americas. Outbreaks of the disease are increasingly occurring among vulnerable populations, with disproportionately involving persons living in poverty and children [1,2]. Contemporarily, case-fatality rates (CFRs) from 20% to 57.5% are now documented in areas where the disease is hyperendemic [2,4,5] that are as high, or higher, than many of other lethal infectious diseases identified by the World Health Organization (WHO), including malaria, tuberculosis, HIV, invasive meningococcal disease, and dengue hemorrhagic fever [6].

**Fig 1:**
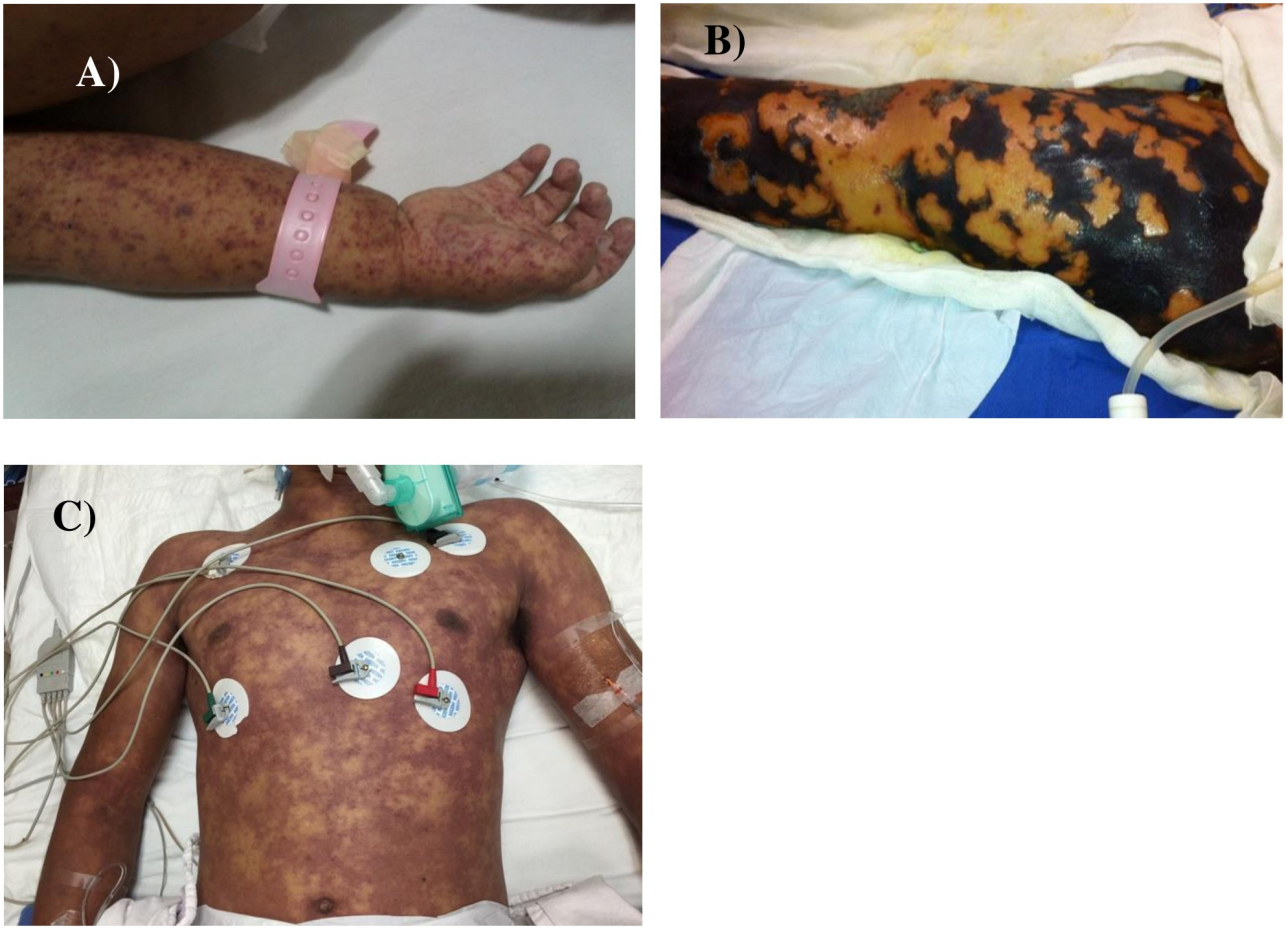
Clinical images of severe Rocky Mountain spotted fever in pediatric patients in northern Mexico. A) Petechiae and ecchymotic lesions on the right upper extremity of a 1-year-old female patient from Sonora, Mexico. B) Extensive cutaneous necrosis on the left lower extremity of a 7-year-old female patient, from Sonora, Mexico. C) Confluent petechial rash involving the thorax, abdomen, and upper extremities of an 18-year-old male patient from Sonora, Mexico.

Another salient and ominous feature of RMSF is the frequency of long-term sequelae experienced among patients who survive severe disease, which can include permanent cognitive deficits and gangrenous loss of digits or appendages (Fig 1) [1]. The daunting morbidity and mortality associated with RMSF can incur enormous medical and indirect costs on low-income communities where this disease is endemic.

Despite underestimation of its magnitude in Latin America, [2] that is already listed as a part of a heterogeneous group of rickettsial diseases of the PLoS neglected tropical diseases (NTD) [7] and although the impact of severe and fatal cases is commensurate with, or exceeds, the metrics of many of other infectious diseases, the RMSF is not recognized by WHO as an NTD [8,9]. An interplay of structural and technical barriers can explain the current status of the disease in the region.

## I. Structural barriers

### A) Poverty and socially disadvantaged populations

Poverty has been identified as a key determinant for most rickettsial diseases [10] and this is particularly evident with RMSF in Mexico and other countries of Latin America where increased tick exposure, higher morbidity, and greater severity of clinical manifestations occur among economically disadvantaged groups. All of that leading to a higher rate of fatal outcomes in people from socially marginalized communities, mainly in Latin American countries [1,2]. Vulnerable populations including people living in poverty, children, pregnant women, migrants, indigenous people, and older adults are even at higher risk of death and long-term RMSF sequelae when compared with other demographic groups in endemic regions [4,11–16].

### B) Neglect of scientific research

Although RMSF was identified and characterized in the early 20th century, its contemporary body of research is limited when compared to other TBDs such as Lyme disease and human anaplasmosis [10]. In addition, a regional gap in scientific publications can be clearly identified and our review of the PubMed database revealed 70% of 1,803 papers concerning RMSF published during 1916 to 2023 were studies predominantly performed in the US, while in Latin America, together with Brazil and Mexico represented 11% of all results for the disease. Although this database is among the largest medical repositories worldwide, it only includes 198 reviews, 2 systematic reviews and just 1 meta-analysis concerning the disease and reveals few multicenter collaborations across the Americas.

### C) Budgetary constraints

The impact of financial shortcomings is summarized in 2 negative effects on magnitude and impact of RMSF. On one hand, it hinders the acquisition of laboratory equipment and reagents to establish a clinically actionable and epidemiologically critical diagnosis, as well as hampering access to intravenous doxycycline and other drugs needed for the management of severe cases, which impedes an accurate estimation of the true incidence rate and contributes to the unacceptable fatality rates observed in some Mexican populations [5,17,18]. On the other hand, economic constraints halts implementation of effective public health measures such as those targeting tick exposure, control of the canine population, sanitary improvement of dwellings, and community-based education campaigns in rural localities and urban slums, which are fundamental for reducing fatal outcomes of RMSF in endemic areas [11,12].

### D) Political support

An example of how important political support is to alleviate the deleterious impact of RMSF, occurred in April 2015 in México. Due to the significant increase in cases and deaths caused by the disease, the Ministry of Health issued a national declaration of epidemiological emergency, which made a call for strengthening epidemiological surveillance, medical care, and prevention of the disease [19]. Among other benefits of this declaration, public hospitals and clinicians had access for the first time to intravenous doxycycline to care for severe cases, and some regions had financial support for dog spaying and neutering campaigns and acquisition of acaricides. Thus, political support is at the heart of the success of national and regional programs for prevention and control of RMSF.

## II. Technical barriers

### A) Weak epidemiological surveillance systems

The geographic distribution of RMSF is restricted to the Americas, with Mexico [2,5,17,18], Brazil [4,13], and the US [1,20] constituting the countries with the heaviest recognized burden, but there is an uncertainty about the actual magnitude and distribution of the disease, as most statistics come from small scientific reports and less from governmental agencies.

The benefits of good epidemiological surveillance systems (ESS) are well accepted for disease tracking and estimation of its impact on defined populations. Mexico has an established national ESS for monitoring the disease. In this system, 5,957 cases of RMSF and spotted fever group rickettsiosis (SFGR) were notified in the 1990 to 2022 period, [21] with the vast majority reported in Mexican states bordering with the US; in this region, vulnerable populations such as children, migrant day laborers, and older adults have been the most affected [2,5,17,18]. Another salient example occurs in Brazil where 1,245 cases were confirmed during the 2007 to 2015 period with the majority being reported from the state of São Paulo [4]; moreover, the CFR increased from 19.2% to 40.2% in different states during the last decade [4,13]. In spite of that, ESS targeting RMSF in Latin America are mostly based on passive models reflecting the occurrence only of notified cases, not actively looking prospectively for risk factors preceding the disease, which may underestimate the true regional burden of morbidity and mortality.

### B) Poor community risk perception

A poor community risk perception of RMSF has been documented regarding exposures, clinical manifestations, and preventive measures [11,12]. Difficult access to information about the relevance of certain risk factors including the distribution of infected ticks has a negative impact on the success of preventive campaigns for tick control and protection of natural and incidental hosts, including pet dogs. Such risk factors include travel history to endemic areas or the role of stray and free-roaming dogs, as well as the contact with wild hosts [1,2].

### C) Insufficient laboratory diagnostic capacity

In several regions of Latin America, laboratory confirmation of *R*. *rickettsii* is not achieved because of (a) limited access to effective diagnostic technology; (b) difficulties to support entomological and epidemiological surveillance systems at the regional and local levels; and (c) funding obstacles that hinder the capacity to detect the pathogen in populations of ticks and hosts [22].

Laboratory methods and technologies to confirm infections with *R*. *rickettsii* form part of a complicated process that requires specific conditions to obtain a valid result either through the indirect immunofluorescence antibody (IFA) assay, which is the current gold standard, or by molecular biology techniques that are not usually available in clinical settings. The insufficient training of laboratory personnel and severe shortcomings of reagents and equipment, decrease the test validity and reliability, thus contributing to the underestimation of the RMSF burden [1,22].

### D) Inadequate physician training

It is pivotal for a better understanding and management of the disease to improve physician’s knowledge, attitudes, and practices (KAP) regarding RMSF. Importantly, several studies have documented medical gaps regarding the rapid use of doxycycline, education about preventive behaviors (i.e., avoiding contact with tick infested dogs, restricting dogs to roam freely), and diagnostic procedures, leading to delay in clinical diagnosis and initiation of treatment, which is associated with fatal outcomes [23,24]. To bridge the gap in medical KAP, efforts need to stress the importance of integrating clinical and epidemiological characteristics of the disease with the understanding of the social determinants of health.

## Conclusions

RMSF is a serious public health problem in several regions across the Americas. Currently, WHO considers neither RMSF nor any other rickettsiosis as an NTD [8], which could perpetuate technical and structural barriers that hamper appropriate consideration of the disease. As noted aptly by Salje and colleagues [10], “There are few infections that are as dangerous but as easy to treat as rickettsioses; therefore, the impact of their neglect on patient health globally is substantial.” We argue that RMSF should be considered as an NTD because its occurrence is largely influenced by socio-environmental determinants that increase the risk of fatalities, particularly for underserved individuals and communities.

## References

[1] Biggs HM, Behravesh CB, Bradley KK, Dahlgreen FS, Drexler N, Dumler JS, et al. Diagnosis and management of tickborne rickettsial diseases: Rocky Mountain spotted fever and other spotted fever group rickettsioses, ehrlichioses, and anaplasmosis—United States. MMWR Recomm Rep. 2016;65:1–44. doi: 10.15585/mmwr.rr6502a1 27172113

[2] Álvarez-Hernández G, Roldán JF, Milan NS, Lash RR, Behravesh CB, Paddock CD. Rocky Mountain spotted fever in Mexico: Past, present, and future. Lancet Infect Dis. 2017:17. doi: 10.1016/S1473-3099(17)30173-1 28365226

[3] Centers for Disease Control and Prevention. Severe and fatal confirmed Rocky Mountain spotted fever among people with recent travel to Tecate, Mexico. [Internet]. Atlanta, US: Emergency and Preparedness Response, 2023 [Accessed: 2024 Jan 17]. https://emergency.cdc.gov/han/2023/han00502.asp.

[4] de Oliveira SV, Guimarães JN, Reckziegel GC, Neves BM, Araújo-Vilges KM, Fonseca LX, et al. An update on the epidemiological situation of spotted fever in Brazil. J Venom Anim Toxins Incl Trop Dis. 2016:22. doi: 10.1186/s40409-016-0077-4 27555867 PMC4994305

[5] Álvarez-López DI, Ochoa-Mora E, Nichols Heitman K, Binder AM, Álvarez-Hernández G, Armstrong PA. Epidemiology and clinical features of Rocky Mountain spotted fever from enhanced surveillance, Sonora, Mexico: 2015–2018. Am J Trop Med Hyg. 2021;104:190–197. doi: 10.4269/ajtmh.20-0854 33146112 PMC7790062

[6] World Health Organization. World health statistics 2022: monitoring health for the SDGs, sustainable development goals. [Internet] Geneva: World Health Organization; 2022 [Accessed: 2024 Jan 9]. https://iris.who.int/bitstream/handle/10665/356584/9789240051140-eng.pdf?sequence=1.

[7] PLoS Neglected Tropical Diseases. Major NTDs within the scope. [Internet] San Francisco US: PLoS Neglected Tropical Diseases; 2024 [Accessed: 2024 Mar 20]. https://journals.plos.org/plosntds/s/journal-information.

[8] World Health Organization. Recommendations for the adoption of additional diseases as neglected tropical diseases. [Internet] Geneve, Switzerland: The WHO Strategic and Technical Advisory Group for Neglected Tropical Diseases (WHO-STAG); 2016 [Accessed: 2023 Sep 1]. https://www.who.int/publications/m/item/ninth-report-of-the-strategic-and-technical-advisory-group-for-neglected-tropical-diseases-(stag-ntds)).

[9] Hotez PJ, Aksoy S, Brindley PJ, Kamhawi S. What constitutes a neglected tropical disease. PLoS Negl Trop Dis. 2020;14(1):e0008001. doi: 10.1371/journal.pntd.0008001 31999732 PMC6991948

[10] Salje J, Weitzel T, Newton PN, Varghese GM, Day N. Rickettsial infections: A blind spot in our view of neglected tropical diseases. PLoS Negl Trop Dis. 2021;15. doi: 10.1371/journal.pntd.0009353 33983936 PMC8118261

[11] Alvarez-Hernandez G, Drexler N, Paddock CD, Licona-Enriquez JD, Delgado-de la Mora J, Straily A, et al. Community-based prevention of epidemic Rocky Mountain spotted fever among minority populations in Sonora, Mexico, using a one health approach. Trans R Soc Trop Med Hyg. 2019;114:293–300. doi: 10.1093/trstmh/trz114 31819997

[12] Drexler N, Miller M, Gerding J, Todd S, Adams L, Dahlgreen FS, et al. Community-based control of the brown dog tick in a region with high rates of Rocky Mountain spotted fever, 2012–2013. PLoS ONE. 2014:9. doi: 10.1371/journal.pone.0112368 25479289 PMC4257530

[13] Amâncio FF, Amorim VD, Chamone TL, de-Brito MG, Calic SB, Leite AC, et al. Epidemiological characteristics of Brazilian spotted fever in Minas Gerais state, Brazil, 2000–2008. Cad Saude Publica. 2011;27:1969–1976. doi: 10.1590/s0102-311x2011001000010 22031201

[14] Tribaldos M, Zaldivar Y, Bermudez S, Samudio F, Mendoza Y, Martinez AA, et al. Rocky Mountain spotted fever in Panama: a cluster description. J Infect Dev Ctries. 2011 Oct 13;5(10):737–741. https://jidc.org/index.php/journal/article/view/21997944/617. doi: 10.3855/jidc.2189 21997944

[15] Licona-Enriquez JD, Delgado de la Mora J, Paddock CD, Ramirez-Rodriguez CA, Candia-Plata MC, Alvarez-Hernandez G. Rocky Mountain spotted fever and pregnancy: four cases from Sonora, Mexico. Am J Trop Med Hyg. 2017;97(3):795–798. doi: 10.4269/ajtmh.16-0917 28722584 PMC5590584

[16] Ponce-Najera E, Lozano-Lazcano V, Ploneda-Gonzalez C, Montoya-Hinojosa M, Gonzalez-Oropeza D. Case report: fatal rickettsiosis in pregnancy. Am J Trop Med Hyg. 2024;110(2):320–322. doi: 10.4269/ajtmh.23-0419 38190746 PMC10859820

[17] Zazueta OE, Armstrong PA, Márquez-Elguea A, Hernández-Milán NS, Peterson AE, Ovalle-Marroquín DF, et al. Rocky Mountain spotted fever in a large Metropolitan Center, Mexico–United States border, 2009–2019. Emerg Infect Dis. 2021:27. doi: 10.3201/eid2706.191662 34014151 PMC8153879

[18] Estrada-Mendizabal RJ, Tamez-Rivera O, Vela EH, Blanco-Murillo P, Alanis-Garza C, Flores-Gouyonnet J, et al. Rickettsial disease outbreak, Mexico, 2022. Emerg Infect Dis. 2023;29(9):1944–1947. doi: 10.3201/eid2909.230344 37610151 PMC10461685

[19] Government of Mexico. Declaratoria de Emergencia Epidemiológica EE-01-2015. [Internet] Mexico; National Center of Preventive Programs and Disease Control (CENAPRECE). 2015 [Accessed: 2023 Sep 5]. http://www.cenaprece.salud.gob.mx/programas/interior/emergencias/descargas/pdf/Declaratoria_Emergencia_EE012015.pdf.

[20] Dahlgren FS, Holman RC, Paddock CD, Callinan LS, McQuiston JH. Fatal Rocky Mountain spotted fever in the United States, 1999–2007. Am J Trop Med Hyg. 2012;86:713–719. doi: 10.4269/ajtmh.2012.11-0453 22492159 PMC3403778

[21] Government of Mexico. Histórico Boletín Epidemiológico. [Internet] México; Dirección General de Epidemiología. Secretaría de Salud. 2022 [Accessed: 2023 Apr 18]. https://www.gob.mx/salud/acciones-y-programas/historico-boletin-epidemiologico.

[22] Paddock CD. Perspectives on the laboratory diagnosis of rickettsial diseases in the 21st Century. Acta Med Costarric. 2013;Suppl 1. https://doaj.org/article/277fe9b6410c47a393741f68e7171dcf.

[23] Zientek J, Dahlgren FS, McQuiston JH, Regan J. Self-reported treatment practices by healthcare providers could lead to death from Rocky Mountain Spotted Fever. J Pediatr. 2014;164:416–418. doi: 10.1016/j.jpeds.2013.10.008 24252781 PMC4699435

[24] Bestul N, Padilla R, Montaño T, Márquez A, Fierro M, Zazueta O, et al. Knowledge, attitudes, and practices on Rocky Mountain spotted fever in a highly endemic region–Mexicali, Mexico. Am J Trop Med Hyg. 2022;107(4):773–779. doi: 10.4269/ajtmh.21-1017 35995132 PMC9651539

